# Length of Stay in In-Patient Rehabilitation after Stroke in Qatar

**DOI:** 10.1100/tsw.2008.81

**Published:** 2008-05-23

**Authors:** Loganathan Venkatachalm, Ana Bobinac Georgievski, Wafaa Al Yazeedi, Rajvir Singh, Hilda Uribazo Garrido

**Affiliations:** ^1^ Hamad Medical Corporation, Doha, Qatar; ^2^ Weill Cornell Medical College, Doha, Qatar; ^3^ Ameijeiras Hospital, Havana, Cuba

**Keywords:** stroke, rehabilitation, length of stay, disability, mobility

## Abstract

The objective of this study was to analyze the factors predicting length of stay in a stroke patient rehabilitation unit at Hamad Medical Corporation (HMC) in Qatar. The medical rehabilitation data of 100 stroke patients discharged from a 15-bed inpatient rehabilitation unit (IPRU) were collected retrospectively from medical records during the period from September 2004 to April 2007. A questionnaire was developed, and variables included in the study were age of the patient, length of stay in acute care (LOSa), length of stay in rehabilitation (LOSr), functional independence measure on admission and discharge (FIMa and FIMd), modified disability scale, and modified mobility scale. Patients were grouped by impairments defined by cause as ischemic or hemorrhagic stroke, and right or left body side deficit. A significant negative correlation was observed between LOSr and FIMa (*r* = −0.44, *p* = 0.00), and positive correlation between LOSr and LOSa (*r* = 0.37, *p* = 0.00). There was no correlation between LOSr and FIMd (*r* = −0.03, *p*= 0.76). We observed that low admission FIMa and FIMd were related to extended LOS in both acute and IPRU. Multivariate regression analysis was performed by taking age, LOSa, cause of hemorrhage or ischemia, and FIMa as independent variables, and LOSr as dependent variable. The model could explain only 26% variation for LOSr. This study supports the hypothesis of an association between LOSr, LOSa, and FIMa. Further research is needed to confirm the results of this and other similar studies.

## INTRODUCTION

Cerebrovascular accident (stroke) is one of the major illnesses causing disability in human beings. With an incidence of 600,000 new cases per year in the U.S., stroke stands out as the third leading cause of death, the leading cause of paralysis, and a major cause of disability[1]. Stroke is also a major health problem in Qatar. The average annual incidence of stroke for 5 years was 11.7/100,000 population[2,3].

Prediction of length of stay (LOS) has become increasingly important for policy makers in health care administration. Fourteen studies found age negatively correlated with function on or after discharge, and four studies showed no correlation[4]. Brosseau et al.[5] reported that the prediction of poststroke rehabilitation LOS also involved the process of functional recovery in stroke patients. Galski et al.[6]

revealed the primary importance of higher-order cognitive impairments in extending LOS. Lin et al.[7] reported that the admission functional independence measure score was useful in the prediction of functional outcome for the stroke survivors following rehabilitation therapy.

Keeping in view all the previously cited variables for LOS and importance of the topic, we decided to conduct a study of LOS in inpatient rehabilitation after stroke in Qatar, where there were no previous studies conducted earlier. The objective of this study was to analyze length of stay in rehabilitation unit (LOSr), and relative importance of available demographic and clinical data for prediction LOSr.

## METHODS

A total of 100 patients with stroke, discharged from the inpatient rehabilitation unit (IPRU) in Hamad Medical Corporation, Doha, Qatar, during the period from September 2004 to April 2007, were included in this study retrospectively. All descriptive data were collected from demographic data files maintained by the office of the Physical Medicine and Rehabilitation Department of the Hamad Medical Corporation. The collected demographic information on age, sex, education, nationality, primary diagnosis, and medical comorbidities have been included in the study.

The functional independence measure (FIM) was used for measuring the functional status on admission and discharge[8]. It analyzed 18 items and seven levels of performance in eating, grooming, bathing, upper and lower body dressing, toileting, bladder and bowel management, bed transfer, toilet transfer, shower transfer, locomotion, stairs, comprehension, expression, social interaction, problem solving, and memory. By summing the points for each item, the possible total score ranges from 18 (lowest) to 126 (highest level of independence). It was also expressed in percentage of the functional levels: less than 25%, total assistance; 25–49%, maximal assistance; 50–74%, moderate assistance; 75% or more, minimal contact assistance; then supervision (cuing, coaxing, prompting), modified independence (extra time, devices), and complete independence (timely, safely)[9].

The FIM was administered to every stroke patient within 78 h of admission to the rehabilitation unit and before discharge by the staff occupational therapist, and was reviewed by rehabilitation team conference. A modified disability scale[10] was used on discharge to evaluate disability level. Mobility outcome was recorded based on patient condition on discharge using the modified mobility scale: no walking deficit, slow gait speed (more than 6 sec/10 m), walks with aid, walks with assistance of other person, self-propel wheelchair, or wheelchair propelled by other.

### Variables

The dependent variable was length of stay in rehabilitation (LOSr) and the independent variables were age, functional independence measure on admission (FIMa), functional independence measure on discharge (FIMd), length of stay in acute care (LOSa), modified disability scale, and modified mobility scale.

### Statistical Methods

Descriptive analysis was done for continuous as well as categorical variables. Pearson correlation coefficient was used to see association between the continuous variables. Student t test and one-way ANOVA with posthoc Boneferroni analysis were performed to see mean level differences between and among categories of different variables, respectively. Regression analysis was performed to see predictor variables for dependent variable (LOSr). A $p$ value of 0.05 was considered to be a statistically significant level. SPSS 14.0 statistical package was used for analysis

## RESULTS

The study included 100 stroke patients with an age range of 19–90 years. The distribution of qualitative characteristics is shown in Table 1. Most of the patients (72%) were expatriates, and the cause of lesion was ischemia in 73% and hemorrhage in 27%. The right side was affected in 54%. In the category of comorbidities, hypertension (35%) was the most common among the total stroke patients.

The majority of stroke patients on discharge were in the disability range of independence in self-care (46%), followed by moderate assistance for daily living (26%). The rest were in two other groups of disability (15%), with up to maximal assistance in daily living and minor symptoms not affecting life style (13%). Regarding the mobility category, most of the patients were in the group of walks with aid (21%), and in the group of slow gait speed and walks with another person to help (20%). The others were in wheelchair propelled by themselves (16%) or by others (17%). Walking was preserved in only 6% of the stroke patients. There were 85% who were employed and 11% retired. Only 8% returned to gainful employment.

The mean levels of age, FIMa, FIMd, LOSa, and LOSr were 50 years, 58%, 78%, 22 days, and 59 days, respectively (Table 2). In 100 patients studied, FIMd was strongly correlated with FIMa (r = 0.631, $p$ = 0.00) and negatively correlated with age and LOSa (r = –0.23, $p$ = 0.02; r = –0.41, $p$ = 0.00, respectively) (Tables 3 and 4, Fig. 1).

LOSr was positively correlated with LOSa (r = 0.37, $p$ = 0.00) and negatively with FIMa (r = –0.44, $p$ = 0.00), whereas there was no correlation with FIMd (r = –0.03, $p$ = 0.760) (Table 5).

LOSr mean levels were compared according to different categories of side affected, cause of hemorrhage or ischemia, modified mobility scale, and modified disability scale (Table 6). Regarding the above categories, the hemorrhagic subgroup patients had significantly longer LOSr than the ischemic subgroup ($p$ = 0.018).

There was an observation that the low FIMa and FIMd were related to extended LOSa and IPRU (r = –0.41, r = –0.44, and r = –0.31) (Tables 3–5 and Figs. 2–4)

A multivariate regression analysis was performed to see the effect of independent variables of age, FIMa, LOSa, and stroke cause on the dependent variable LOSr (Table 7). FIMa and LOSa were found to be statistically significant variables. The model summary showed that these four predictors could explain only a 26% variation for dependent variable. Results of the multivariate analysis are presented in Table 8.

## DISCUSSION

Qatar is a small country with an area of 11,427 km$^2$, extending into the Persian Gulf from the eastern Arabian Peninsula. The entire country is served by the Hamad Medical Corporation[2]. All patients with stroke episodes in Qatar are admitted to the Hamad General Hospital for acute care management and are then transferred, after becoming medically stable, to the stroke unit in Rumailah Hospital for subacute care and rehabilitation management.

Stroke, a leading cause of disability, necessitates the expenditure of considerable resources for the rehabilitation of these patients. Many clinical studies on predictors of functional outcome after stroke have been published in the world literature, but this present study is a pioneering study of functional outcome after stroke, in Qatar. Cifu and Stewart[11] cited that rehabilitation outcome was defined as functional abilities at rehabilitation discharge and at follow-up (typically 3 or 12 months). They also reported that there was medical literature from 1950 to 1998 that revealed that rehabilitation interventions have a variable relationship with functional outcome after stroke[11]. The prediction of inpatient rehabilitation outcome is relevant for rehabilitation specialists to maximize their client's preparation for a return home.

**TABLE 1**
**Distribution of Qualitative Characteristics in the Sample (100)**

| Variables | Category | N |
|---|---|---|
| Status | Qatari | 14 |
| | Qatari resident | 14 |
| | Expatriate | 72 |
| Side of body affected | Right | 54 |
| | Left | 46 |
| Cause | Hemorrhage | 27 |
| | Ischemia | 73 |
| Morbidity | HTN | 35 |
| | DM | 16 |
| | CAD | 3 |
| | HF | 5 |
| | HTN and DM | 1 |
| | HTN, DM, and CAD | — |
| | HTN, DM, and Asth | — |
| | Psychological problem | 20 |
| | Asthma | 4 |
| | Epilepsy | 4 |
| | DM and CAD | 1 |
| | HTN, DM, and epilepsy | 1 |
| | No comorbidity | 10 |
| Occupation (ICF)* | Paid employment | 85 |
| | Self-employed | 2 |
| | Volunteer | — |
| | Student | — |
| | Housemaker | — |
| | Retired | 11 |
| | Unemployed | 2 |
| | Others | — |
| Martial status (ICF)* | Single | 9 |
| | Married | 88 |
| | Separated | 1 |
| | Divorced | 2 |
| Disability scale** on discharge | Well | — |
| | Minor symptom not affecting life style | 13 |
| | Disabled, but independent self-care | 46 |
| | Disabled, with up to moderate assistance | 26 |
| | Disabled, with up to maximal assistance | 15 |
| | Total assistance | — |
| Mobility scale on discharge | No walking deficit | 6 |
| | Slow gait speed | 20 |
| | Walks with aid | 21 |
| | Walks with help of other person | 20 |
| | Wheelchair, self-propel | 16 |
| | Wheelchair, propelled by others | 17 |
| Back to work | No | 75 |
| | Yes | 8 |
| | Sick leave | 17 |

Asth, Asthma; HTN, hypertension; DM, Diabetes Mellitus; CAD, Coronary Arterial Disease; HF, Heart failure.

* ICF check list of the World Health Organization, September 2003.

** Adopted from Scottish Intercollegiate guidelines network, 2003 (www.sign.ac.uk)

**TABLE 2**
**Descriptive Statistics of Included Variables in the Study**

| | Mean ± SD | Minimum | Maximum |
|---|---|---|---|
| Age (years) | 50 ± 10.48 | 19.0 | 90.00 |
| FIMa (%) | 58 ± 17.8 | 20.00 | 93.00 |
| FIMd (%) | 76 ± 16.7 | 25.00 | 99.00 |
| LOSa (days) | 22 ± 15.8 | 6.00 | 91.00 |
| LOSr (days) | 59 ± 38.7 | 6.00 | 218.00 |

**TABLE 3**
**Association of FIMd with Different Variables in the Study**

| Variables | r | *p* (Two Tailed) |
|---|---|---|
| LOSa | –0.33 | 0.01 |
| Age | –0.23 | 0.22 |

**TABLE 4**
**Association of FIMa with Different Variables in the Study**

| Variables | r | *p* (Two Tailed) |
|---|---|---|
| FIMd | 0.631 | 0.00 |
| LOSa | –0.41 | 0.00 |

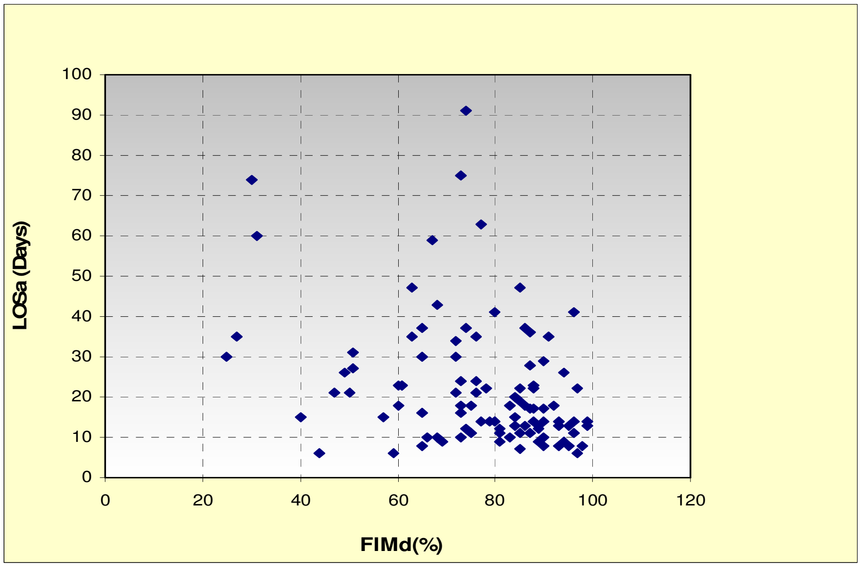

**FIGURE 1**. Correlation of LOSa (days) and FIMd (%).

**TABLE 5**
**Association of LOSr with Different Variables in the Study**

| Variables | r | *p* (Two Tailed) |
|---|---|---|
| FIMd | –0.03 | 0.760 |
| FIMa | –0.44 | 0.00 |
| LOSa | 0.37 | 0.00 |

**TABLE 6**
**Comparison of LOSr Mean Levels According to Different Categories in this Study**

| Variables | Category | N | Mean ± SD (LOSr) | Sig(*p*) |
|---|---|---|---|---|
| Affected side | Right | 54 | 54.9 ± 28.07 | 0.206 |
| | Left | 46 | 64.7 ± 48.1 | |
| Stroke cause | Hemorrhage | 27 | 74.4 ± 39.14 | 0.018 |
| | Infarction | 73 | 53.9 ± 37.35 | |
| Modified mobility scale | 0 | 6 | 37.16 ± 19.83 | 0.385 |
| | 1 | 20 | 61.15 ± 48.73 | |
| | 2 | 21 | 68.85 ± 41.76 | |
| | 3 | 20 | 65.25 ± 41.02 | |
| | 4 | 16 | 58.68 ± 30.06 | |
| | 5 | 17 | 47.58 ± 28.60 | |
| Modified disability scale | 0 | — | | 0.064 |
| | 1 | 13 | 38.07 ± 22.91 | |
| | 2 | 46 | 63.54 ± 38.07 | |
| | 3 | 26 | 54.26 ± 35.60 | |
| | 4 | 15 | 74.40 ± 49.66 | |
| | 5 | — | | |

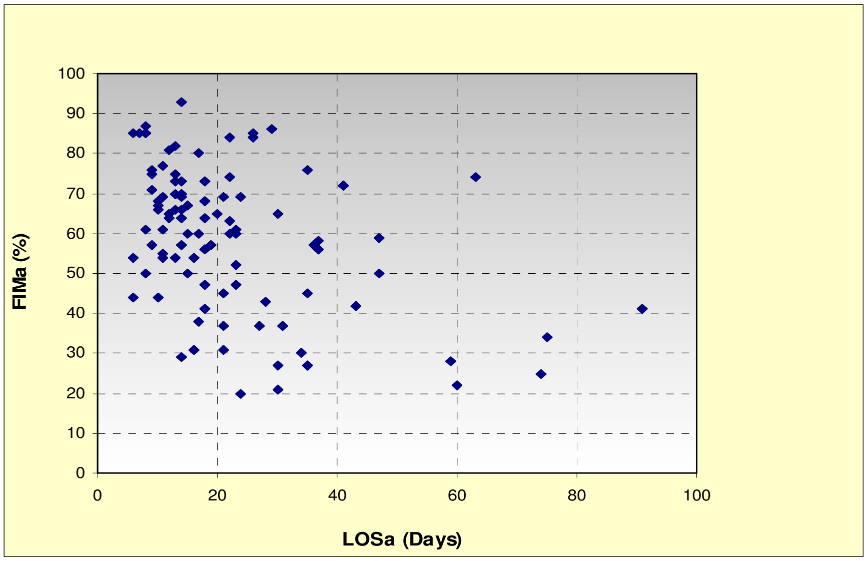

**FIGURE 2.** Correlation of FIMa (%) and LOSa (days).

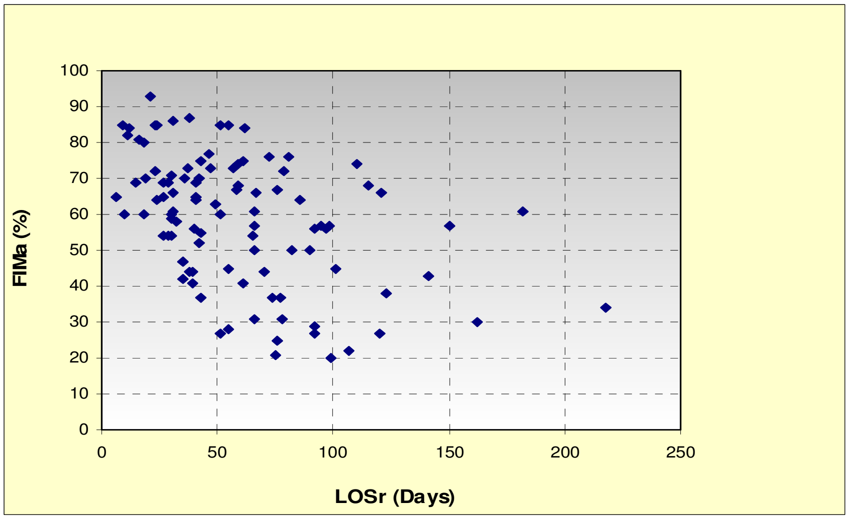

**FIGURE 3**. Correlation of FIMa (%) and LOSr (days).

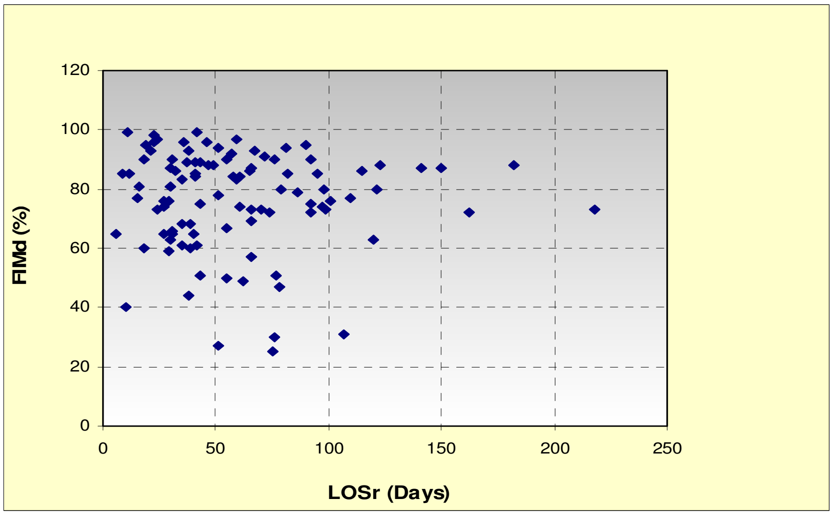

**FIGURE 4.** Correlation of FIMd (%) and LOSr (days).

**TABLE 7**
**Multivariate Regression Analysis on LOSr with Different Independent Variables**

| Model | R | R Square | Adjusted R Square | Std. Error of the Estimate |
|---|---|---|---|---|
| 1 | 0.507* | 0.257 | 0.226 | 34.09191 |

* Predictor: (Constant), Age, LOSa, Cause, FIMa. Dependent variable: LOSr.

**TABLE 8**
**Coefficients***

| Model | Unstandardized Coefficients | | Sig(*p*) |
|---|---|---|---|
| | B | Std. Error | |
| 1 Constant | 112.74 | 26.761 | 0.000 |
| FIMa | –0.749 | 0.228 | 0.001 |
| LOSa | 0.541 | 0.237 | 0.025 |
| Cause | 3.872 | 8.668 | 0.656 |
| Age | –0.452 | 0.345 | 0.193 |

* Dependent variable: LOSr.

The aim of our study was to analyze the factors that can predict the rehabilitation outcome of patients after stroke. Older stroke patients had slow functional recovery, whereas younger patients had better prognosis. We found in our study that the age was correlated negatively with FIMd, which was a confirmation of earlier studies[1,5,12,13,14,15,16,17]. Others found that increased incidence of chronic illness (diabetes, coronary disease, and hypertension) partially explains the negative correlation between age and FIMd in our older patients[1,4].

Previous studies[1,12] reported that onset-admission interval (OAI), the number of days from onset of stroke to rehabilitation admission (similar to LOSa), was unrelated to the severity of weakness on admission and unrelated to length of stay, but was related to discharge disposition and functional status on discharge. We found that FIMd was strongly correlated with FIMa and negatively correlated with LOSa ($r = –0.23$), which concurred with the reports. This study not only shows the relationship between early admission to the hospital and FIM on discharge from hospital, but also indicates the early benefit of rehabilitation.

Inouye et al.[1] reported no difference in functional recovery related to nature of stroke. However, Paolucci et al.[18] and Kelly et al.[19] reported that hemorrhagic stroke patients had better functional recovery. Regarding comparison study of LOSr with cause of hemorrhage or ischemia, Paolucci et al.[18] reported that no difference was found, whereas Kelly et al.[19] reported a longer recovery in the hemorrhagic subgroup. Our study found that the mean LOSr was significantly longer in the hemorrhagic subgroup compared with the ischemic stroke subgroup ($p = 0.018$).

Our study also showed that the side of the lesion was not a significant predictor with LOSr, consistent with studies of Kotila et al.[13] and Jongbloed[4]. In Qatar, most of the stroke patients were expatriates (72%) living alone. Patients living alone stayed in the hospital significantly more often than those who had families. It seems that family involvement is an important predictor of disposition of the stroke patient. Early involvement of social services, in provision of environmental aids and adaptations or placement in residential care, would probably reduce the unnecessary hospitalization[16].

From our study, we understand that some barriers are affecting prompt discharge and they should be explored to distinguish between patient's readiness and any restrictions from administrative and social units. These could help to explain a larger percentage of variance in the intermediate and rehabilitation LOS[5]. In relation to other findings, overall, our study showed that FIM admission was the best predictor of functional outcome in stroke inpatients[7,14], and also stroke patients of lower admission and discharge FIMs were related to extended LOS in both acute and IPRU. However, our statistical analysis indicated that only 26% of variation for LOSr was explained by the predictors of age, LOSa, cause, and FIMa. We found that some higher FIMd was also associated with extended LOSr (Fig. 1), but only 12% of the total stroke patients. The reason for this extended LOSr might be dependent upon socioeconomic factors. Our study identified the need for further prospective studies in large stroke populations in order to evaluate other predictors influencing LOS in rehabilitation units.

## ACKNOWLEDGMENTS

We are very thankful and grateful to Professor Leopold Streletz, Department of Medicine, Weill Cornell Medical College, Qatar who edited the manuscript. We are thankful to the occupational therapists, physiotherapists, and nurses of the stroke unit for their dedicated contribution to the rehabilitation process and are grateful and thankful to the Medical Research Committee, Hamad Medical Corporation, for permission to conduct this study.
